# Update from a cohort study for birth defects in Hunan Province, China, 2010–2020

**DOI:** 10.1038/s41598-023-47741-1

**Published:** 2023-11-20

**Authors:** Xu Zhou, Shenglan Cai, Hua Wang, Junqun Fang, Jie Gao, Haiyan Kuang, Donghua Xie, Jian He, Aihua Wang

**Affiliations:** 1 Hunan Provincial Maternal and Child Health Care Hospital, Changsha, Hunan China; 2 The Hunan Children’s Hospital, Changsha, Hunan China; 3 National Health Commission Key Laboratory of Birth Defects Research, Prevention and Treatment, Hunan Provincial Maternal and Child Health Care Hospital, Changsha, Hunan China

**Keywords:** Epidemiology, Genetics research

## Abstract

To define the relationship between sex, residence, maternal age, and a broad range of birth defects by conducting a comprehensive cross-analysis based on up-to-date data. Data were obtained from the Birth Defects Surveillance System in Hunan Province, China, 2010–2020. Prevalences of birth defects (number of cases per 10,000 fetuses (births and deaths at 28 weeks of gestation and beyond)) with 95% confidence intervals (CI) were calculated by sex, residence, maternal age, year, and 23 specific defects. Cross-analysis of sex, residence, and maternal age was conducted, and crude odds ratios (ORs) were calculated to examine the association of each maternal characteristic with birth defects. A total of 1,619,376 fetuses and 30,596 birth defects were identified. The prevalence of birth defects was 188.94/10,000 (95% CI 186.82–191.05). Birth defects were more frequent in males than females (210.46 vs. 163.03/10,000, OR = 1.30, 95% CI 1.27–1.33), in urban areas than in rural areas (223.61 vs. 162.90/10,000, OR = 1.38, 95% CI 1.35–1.41), and in mothers ≥ 35 than mothers 25–29 (206.35 vs. 187.79/10,000, OR = 1.10, 95% CI 1.06–1.14). Cross-analysis showed that the prevalence of birth defects was higher in urban females than in rural males (194.53 vs. 182.25/10,000), the difference in prevalence between males and females was more significant for maternal age < 20 compared to other age groups (OR = 1.64, 95% CI 1.37–1.95), and the prevalence difference between urban and rural areas is more significant for maternal age 25–34 compared to other age groups (OR = 1.49, 95% CI 1.43–1.57). Cleft palates were more frequent in males, and nine specific defects were more frequent in females. Five specific defects were more frequent in rural areas, and eight were more frequent in urban areas. Compared to mothers 25–29, five specific defects were more frequent in mothers < 20, seven specific defects were more frequent in mothers 20–24, two specific defects were more frequent in mothers 30–34, and ten specific defects were more frequent in mothers ≥ 35. Our data indicate that sex, residence, and maternal age differences in the prevalences of birth defects and most specific defects are common. We have found some new epidemiological characteristics of birth defects using cross-analysis, such as residence is the determining factor for the prevalence of birth defects, the difference in prevalence between males and females was more significant for maternal age < 20 compared to other age groups, the prevalence difference between urban and rural areas is more significant for maternal age 25–34 compared to other age groups. And differences in the epidemiological characteristics of some specific defects from previous studies. Future studies should examine mechanisms. Our findings contributed to clinical counseling and advancing research on the risk factors for birth defects.

## Introduction

Birth defects are structural or functional anomalies at or before birth^1^. The accepted prevalence of birth defects is about 2–3% worldwide^2^. Birth defects are associated with many adverse pregnancy outcomes, such as preterm birth, stillbirths, and child mortality^3–5^. WHO estimated that about 12.6% of neonatal deaths worldwide each year are related to birth defects^6^, and 240,000 newborns worldwide die each year from birth defects within the first 28 days of life, and birth defects cause 170,000 children deaths between the ages of 1 month and 5 years^7^. Birth defects have been a significant problem for health care in terms of the resources they require because of their longer life expectancy, especially in low- and middle-income countries^2, 7^. Therefore, the study on birth defects is significant and deserves more attention.

Instead, there have been fewer national studies on birth defects in China recently. There have been some studies on birth defects in Hunan Province, China. E.g., study on the association between ambient air pollution and birth defects (2014–2016)^8^; the epidemiology of chromosomal abnormalities (2016–2019)^9^; the characteristics of the prenatal diagnosis of birth defects and termination of pregnancy for fetal anomalies (2015–2018)^10^; the prevalence of birth defects between 2005 and 2014^11^. However, no studies have analyzed the relationship between birth defects and sex, residence, and maternal age in depth. In addition, many of the previous studies had data limitations or needed to be updated. Therefore, an update from a cohort study for birth defects is needed.

Although in many birth defect cases, the cause is still unknown, many researchers believe that birth defects may result from hereditary polygenic defects or a gene-environment interaction^12, 13^. Risk factors for birth defects may change over time and vary between regions and populations. Epidemiological studies on birth defects help advance research on the risk factors for birth defects. Previous studies have shown that many risk factors were associated with birth defects, such as environmental factors (e.g. chemical toxicants, infection agents, maternal disease, and exogenous factors), genetic causes (e.g. genetic chromosomal aberrations and dysgeneses), and socioeconomic factors^12, 14^. Among them, sex, residence, and maternal age are the most common epidemiological characteristics, which can also be used as proxies for additional epidemiological characteristics, such as healthcare and economic conditions. There were some previous studies on the relationship between sex, residence, maternal age, and birth defects. In general, birth defects were more common in males, urban areas, or fetuses of advanced maternal age^15–18^. However, there are also limitations in these studies. First, some studies only analyzed the prevalence of all birth defects overall, not by disease type. Second, some studies were limited in data, such as relatively few cases included or surveys conducted in unrepresentative districts or hospitals. Third, some studies needed to be updated. Fourth, there is a lack of in-depth analysis methods, such as cross-analysis, in most studies. In addition, some studies have different findings that diverge. E.g., Ahn et al. found that very low-quality evidence suggests that women in the older maternal age group increased the risk of birth defects^19^, and Goetzinger et al. found that advanced maternal age was associated with an overall decreased risk for major anomalies^20^. Xiong et al. found a higher prevalence of birth defects in rural than urban areas^21^. It is essential to conduct systematic research using the latest representative data.

Therefore, we conducted a comprehensive cross-analysis based on the data from the Birth Defects Surveillance System in Hunan Province, south-central China (from 2010 to 2020), to define the relationship between sex, residence, maternal age, and a broad range of birth defects (including 23 types of specific defects in detail). Our study is an essential update on birth defects research. It will provide additional information on birth defects, such as epidemiological characteristics of various specific defects, cross-risk factors for birth defects, etc. Our study may contribute to clinical counseling and advancing research on the risk factors for birth defects.

## Methods

### Data sources

This study used data from the Birth Defects Surveillance System in Hunan Province, China, 2010–2020, which is run by the Hunan Provincial Health Commission and involves 52 representative registered hospitals in Hunan Province. Surveillance data of fetuses (births and deaths at 28 weeks of gestation and beyond) and all birth defects (between 28 weeks of gestation and seven days after delivery) included demographic characteristics such as sex, residence (living in an urban area for more than six months prior to pregnancy is defined as urban, otherwise defined as rural), maternal age, and other key information. The 52 hospitals are required to report all cases of birth defects at birth and at 28 weeks of gestation and beyond in medical records to the Birth Defects Surveillance System.

According to the WHO International Classification of Diseases (Ninth Revision, ICD-9), birth defects were classified into 23 subtypes: anencephaly (Q00), spina bifida (Q05), encephalocele (Q01), hydrocephalus (Q03), cleft palate (Q35), cleft lip (Q36), cleft lip-palate (Q37), anotia/microtia (Q17.2, Q16.0), other external ear defects (Q17), esophageal atresia (Q39), anal atresia (Q42), hypospadias (Q54), bladder exstrophy (Q64.1), talipes equinovarus (Q66.0), polydactyly (Q69), syndactyly (Q70), limb reduction (Q71, Q72), diaphragmatic hernia (Q79.0), omphalocele (Q79.2), gastroschisis (Q79.3), conjoined twins (Q89.4), Down syndrome (Q90), congenital heart defects (Q20-26) or ‘other’ (Q00-Q99, excluding the codes mentioned above).

### Informed consents

We confirmed that informed consent was obtained from all subjects and/or their legal guardian(s). Doctors obtain consent from pregnant women before collecting surveillance data, witnessed by their families and the heads of the obstetrics or neonatal departments. Doctors obtain consent from their parents or guardians for live births, witnessed by their families and the heads of the obstetrics or neonatal departments. Since the Health Commission of Hunan Province collects those data, and the government has emphasized the privacy policy in the “Maternal and Child Health Monitoring Manual in Hunan Province”, there is no additional written informed consent.

### Ethics guideline statement

The Medical Ethics Committee of Hunan Provincial Maternal and Child Health Care Hospital approved the study. (NO: 2022-S013). It is a retrospective study of medical records. All data generated or analyzed during this study were from the Birth Defects Surveillance System. All data were fully anonymized before we accessed them. Moreover, we de-identified the patient records before analysis. We confirmed that all experiments were performed following relevant guidelines and regulations.

### Data quality control

To carry out surveillance, the Hunan Provincial Health Commission formulated the "Maternal and Child Health Monitoring Manual in Hunan Province". Data were collected and reported by experienced doctors. To reduce integrity and information error rates, the Hunan Provincial Health Commission asked the technical guidance departments to conduct comprehensive quality control each year.

### Statistical analysis

The prevalence of birth defects is defined as the number of birth defects per 10,000 fetuses. Prevalences and 95% confidence intervals (CI) were calculated for any defect and 23 specific defects. If the number of birth defects was tiny (≤ 50), we calculated the 95%CI by the Robust Poisson method; if the number of birth defects was > 50, we calculated the 95%CI by the log-binomial method. Chi-square trend tests (χ^2^_trend_) were used to determine trends in prevalence by year. Crude odds ratios (ORs) were calculated to examine the association of each maternal characteristic with birth defects.

Statistical analyses were performed using SPSS 18.0 (IBM Corp., NY, USA).

## Results

### Prevalence of all birth defects and specific defects

Our study included 1,619,376 fetuses, and 30,596 fetuses had at least one birth defect diagnosis. The prevalence of birth defects was 188.94/10,000 (95% CI 186.82–191.05). From 2010 and 2020, the prevalences of birth defects were 187.28, 227.81, 204.96, 189.61, 221.87, 218.39, 182.03, 179.96, 163.14, 160.34, 164.75 per 10,000 fetuses, respectively, and showed a decreasing trend (χ^2^_trend_ = 246.44, P < 0.01) (Table 1).

**Table 1:** Prevalence of birth defects in Hunan province, China, 2010–2020.

Year	Number of fetuses (n)	Number of birth defects (n)	Prevalence (1/10,000)	95% CI (1/10,000)
2010	98,624	1847	187.28	178.74–195.82
2011	107,500	2449	227.81	218.79–236.84
2012	125,583	2574	204.96	197.05–212.88
2013	135,645	2572	189.61	182.28–196.94
2014	143,640	3187	221.87	214.17–229.58
2015	160,629	3508	218.39	211.16–225.62
2016	170,688	3107	182.03	175.63–188.43
2017	196,316	3533	179.96	174.03–185.90
2018	177,762	2900	163.14	157.20–169.08
2019	164,840	2643	160.34	154.22–166.45
2020	138,149	2276	164.75	157.98–171.52
Total	1,619,376	30,596	188.94	186.82–191.05

*CI* confidence intervals.

Table 2 shows the prevalence (95% CI) of each specific defect. The primary specific defects were as follows: congenital heart defects (9779 cases, 60.39/10,000), polydactyly (3185 cases, 19.67/10,000), other external ear defects (2367 cases, 14.62/10,000), talipes equinovarus (1226 cases, 7.57/10,000), and syndactyly (1040 cases, 6.42/10,000) (Table 2).

**Table 2:** Prevalence of specific defects.

Types	Number of fetuses (n)	Birth defects (n)	Prevalence (1/10,000)	95% CI (1/10,000)
Congenital heart defect	1,619,376	9779	60.39	59.19–61.58
Polydactyly	1,619,376	3185	19.67	18.99–20.35
Other external ear defects	1,619,376	2367	14.62	14.03–15.21
Talipes equinovarus	1,619,376	1226	7.57	7.15–7.99
Syndactyly	1,619,376	1040	6.42	6.03–6.81
Hypospadias	1,619,376	865	5.34	4.99–5.70
Cleft lip-palate	1,619,376	777	4.80	4.46–5.14
Hydrocephalus	1,619,376	565	3.49	3.20–3.78
Limb reduction	1,619,376	508	3.14	2.86–3.41
Cleft lip	1,619,376	447	2.76	2.50–3.02
Cleft palate	1,619,376	446	2.75	2.50–3.01
Anal atresia	1,619,376	440	2.72	2.46–2.97
Anotia/microtia	1,619,376	372	2.30	2.06–2.53
Down syndrome	1,619,376	242	1.49	1.31–1.68
Spina bifida	1,619,376	187	1.15	0.99–1.32
Diaphragmatic hernia	1,619,376	162	1.00	0.85–1.15
Omphalocele	1,619,376	149	0.92	0.77–1.07
Esophageal atresia	1,619,376	108	0.67	0.54–0.79
Gastroschisis	1,619,376	103	0.64	0.51–0.76
Anencephaly	1,619,376	70	0.43	0.33–0.53
Encephalocele	1,619,376	53	0.33	0.24–0.42
Bladder exstrophy	1,619,376	24	0.15	0.10–0.22
Conjoined twins	1,619,376	5	0.03	0.01–0.07
Other	1,619,376	11,310	69.84	68.55–71.13

*CI* confidence intervals.

### Prevalence of birth defects by sex, residence, and maternal age

Overall, birth defects were more frequent in males than females (210.46 vs. 163.03/10,000, OR = 1.30, 95% CI 1.27–1.33), in urban areas than in rural areas (223.61 vs. 162.90/10,000, OR = 1.38, 95% CI 1.35–1.41), and in mothers ≥ 35 compared to mothers 25–29 (206.35 vs. 187.79/10,000, OR = 1.10, 95% CI 1.06–1.14). Male fetuses, urban residents, and mothers ≥ 35 were risk factors for birth defects (Table 3).

**Table 3:** Prevalence of birth defects by sex, residence, and maternal age.

Indicator	Number of fetuses (n)	Birth defects (n)	Prevalence (1/10,000, 95% CI)	OR (95% CI)
Sex				
Male	857,109	18,039	210.46 (207.39–213.53)	1.30 (1.27–1.33)
Female	762,018	12,423	163.03 (160.16–165.89)	Reference
Unknown	249	134	–	–
Residence				
Urban	694,501	15,530	223.61 (220.10–227.13)	1.38 (1.35–1.41)
Rural	924,875	15,066	162.90 (160.30–165.50)	Reference
Maternal age (years old)				
< 20	27,319	546	199.86 (183.10–216.63)	1.07 (0.98–1.16)
20–24	339,120	6241	184.04 (179.47–188.60)	0.98 (0.95–1.01)
25–29	693,209	13,018	187.79 (184.57–191.02)	Reference
30–34	385,022	7186	186.64 (182.32–190.95)	0.99 (0.97–1.02)
≥ 35	174,706	3605	206.35 (199.61–213.08)	1.10 (1.06–1.14)

*CI* confidence intervals, *OR* odds ratio.

The results of the cross-analysis of sex, residence, and maternal age were similar to the overall (Tables 4, 5 and 6). There are some new features for cross-analysis. First, the prevalence of birth defects was higher in urban females than in rural males (194.53 vs. 182.25/10,000) (Table 4). It indicates that residence is the main determinant. Second, the OR values of male prevalence to female prevalence in maternal age < 20, 20–24, 25–29, 30–34, and ≥ 35 were 1.64, 1.20, 1.33, 1.32, and 1.27, respectively (Table 5). It indicates that the difference in prevalence between males and females was more significant for maternal age < 20 compared to other age groups. Third, the OR values of urban prevalence to rural prevalence in maternal age < 20, 20–24, 25–29, 30–34, and ≥ 35 were 1.17, 1.22, 1.45, 1.49, and 1.25, respectively (Table 6). It indicates that the prevalence difference between urban and rural areas is more significant for maternal age 25–34 compared to other age groups.

**Table 4:** Prevalence of birth defects by sex cross residence.

Residence	Sex	Number of fetuses (n)	Birth defects (n)	Prevalence (1/10,000, 95% CI)	OR (95% CI)
Urban	Male	368,598	9136	247.86 (242.78–252.94)	1.28 (1.24–1.32)
	Female	325,807	6338	194.53 (189.74–199.32)	Reference
Rural	Male	488,511	8903	182.25 (178.46–186.03)	1.31 (1.27–1.36)
	Female	436,211	6085	139.50 (135.99–143.00)	Reference

*CI* confidence intervals, *OR* odds ratio.

**Table 5:** Prevalence of birth defects by sex cross maternal age.

Maternal age (years old)	Male			Female (reference)			OR (95% CI)
	Total (n)	BD (n)	Prevalence (1/10,000, 95% CI)	Total (n)	BD (n)	Prevalence (1/10,000, 95% CI)	
< 20	14,184	345	243.23 (217.57–268.90)	13,124	197	150.11 (129.15–171.07)	1.64 (1.37–1.95)
20–24	177,481	3527	198.73 (192.17–205.28)	161,594	2681	165.91 (159.63–172.19)	1.20 (1.14–1.26)
25–29	366,384	7736	211.14 (206.44–215.85)	326,734	5217	159.67 (155.34–164.00)	1.33 (1.28–1.38)
30–34	205,528	4295	208.97 (202.72–215.22)	179,435	2865	159.67 (153.82–165.51)	1.32 (1.25–1.38)
≥ 35	93,532	2136	228.37 (218.69–238.06)	81,131	1463	180.33 (171.09–189.57)	1.27 (1.19–1.36)

*BD* birth defect, *CI* confidence intervals, *OR* odds ratio.

**Table 6:** Prevalence of birth defects by residents cross maternal age.

Maternal age (years old)	Urban			Rural (reference)			OR (95% CI)
	Total (n)	BD (n)	Prevalence (1/10,000, 95% CI)	Total (n)	BD (n)	Prevalence (1/10,000, 95% CI)	
< 20	6779	152	224.22 (188.58–259.87)	20,540	394	191.82 (172.88–210.76)	1.17 (0.97–1.42)
20–24	106,323	2231	209.83 (201.13–218.54)	232,797	4010	172.25 (166.92–177.58)	1.22 (1.16–1.29)
25–29	302,455	6868	227.08 (221.70–232.45)	390,754	6150	157.39 (153.45–161.32)	1.45 (1.40–1.50)
30–34	188,465	4220	223.91 (217.16–230.67)	196,557	2966	150.90 (145.47–156.33)	1.49 (1.43–1.57)
≥ 35	90,479	2059	227.57 (217.74–237.40)	84,227	1546	183.55 (174.40–192.70)	1.25 (1.17–1.33)

*BD* birth defect, *CI* confidence intervals, *OR* odds ratio.

### Prevalence of specific defects by sex

Males were more likely to have the following specific defects: congenital heart defect (60.75 vs. 58.04/10,000, OR = 1.05, 95% CI 1.01–1.09), polydactyly (23.66 vs. 15.04/10,000, OR = 1.57, 95% CI 1.46–1.69), other external ear defects (15.47 vs. 13.66/10,000, OR = 1.13, 95% CI 1.04–1.23), syndactyly (7.06 vs. 5.62/10,000, OR = 1.26, 95% CI 1.11–1.42), hypospadias (9.70 vs. 0.05/10,000, OR = 184.88, 95% CI 69.22–493.77), cleft lip, palate (5.19 vs. 4.24/10,000, OR = 1.22, 95% CI 1.06–1.41), cleft lip (3.13 vs. 2.18/10,000, OR = 1.44, 95% CI 1.18–1.74), anal atresia (3.44 vs. 1.59/10,000, OR = 2.17, 95% CI 1.75–2.68) and anotia/microtia (2.70 vs. 1.68/10,000, OR = 1.60, 95% CI 1.29–1.99). The prevalence of cleft palate was lower in males than females (1.93 vs. 3.45/10,000, OR = 0.56, 95% CI 0.46–0.68). It is the only specific defect that is lower in males than females (Table 7).

**Table 7:** Prevalence of specific defects by sex.

Types	Male (total = 857,109)		Female (total = 762,018) (reference)		OR (95% CI)
	BD (n)	Prevalence (1/10,000, 95% CI)	BD (n)	Prevalence (1/10,000, 95% CI)	
Congenital heart defect	5207	60.75 (59.10–62.40)	4423	58.04 (56.33–59.75)	1.05 (1.01–1.09)
Polydactyly	2028	23.66 (22.63–24.69)	1146	15.04 (14.17–15.91)	1.57 (1.46–1.69)
Other external ear defects	1326	15.47 (14.64–16.30)	1041	13.66 (12.83–14.49)	1.13 (1.04–1.23)
Talipes equinovarus	647	7.55 (6.97–8.13)	570	7.48 (6.87–8.09)	1.01 (0.90–1.13)
Syndactyly	605	7.06 (6.50–7.62)	428	5.62 (5.08–6.15)	1.26 (1.11–1.42)
Hypospadias	831	9.70 (9.04–10.35)	4	0.05 (0.01–0.13)	184.88 (69.22–493.77)
Cleft lip-palate	445	5.19 (4.71–5.67)	323	4.24 (3.78–4.70)	1.22 (1.06–1.41)
Hydrocephalus	309	3.61 (3.20–4.01)	254	3.33 (2.92–3.74)	1.08 (0.92–1.28)
Limb reduction	277	3.23 (2.85–3.61)	206	2.70 (2.33–3.07)	1.20 (1.00–1.43)
Cleft lip	268	3.13 (2.75–3.50)	166	2.18 (1.85–2.51)	1.44 (1.18–1.74)
Cleft palate	165	1.93 (1.63–2.22)	263	3.45 (3.03–3.87)	0.56 (0.46–0.68)
Anal atresia	295	3.44 (3.05–3.83)	121	1.59 (1.30–1.87)	2.17 (1.75–2.68)
Anotia/microtia	231	2.70 (2.35–3.04)	128	1.68 (1.39–1.97)	1.60 (1.29–1.99)
Down syndrome	121	1.41 (1.16–1.66)	120	1.57 (1.29–1.86)	0.90 (0.70–1.15)
Spina bifida	84	0.98 (0.77–1.19)	93	1.22 (0.97–1.47)	0.80 (0.60–1.08)
Diaphragmatic hernia	90	1.05 (0.83–1.27)	68	0.89 (0.68–1.10)	1.18 (0.86–1.61)
Omphalocele	76	0.89 (0.69–1.09)	73	0.96 (0.74–1.18)	0.93 (0.67–1.28)
Esophageal atresia	58	0.68 (0.50–0.85)	50	0.66 (0.49–0.86)	1.03 (0.71–1.51)
Gastroschisis	59	0.69 (0.51–0.86)	44	0.58 (0.42–0.77)	1.19 (0.81–1.76)
Anencephaly	29	0.34 (0.23–0.49)	28	0.37 (0.24–0.53)	0.92 (0.55–1.55)
Encephalocele	26	0.30 (0.20–0.44)	27	0.35 (0.23–0.51)	0.86 (0.50–1.47)
Bladder exstrophy	14	0.16 (0.09–0.27)	10	0.13 (0.06–0.24)	1.24 (0.55–2.80)
Conjoined twins	1	0.01 (0.001–0.07)	3	0.04 (0.008–0.12)	0.30 (0.03–2.85)
Other	6889	80.37 (78.48–82.27)	4314	56.61 (54.92–58.30)	1.42 (1.37–1.48)

There were some specific defects with unknown sex.

*BD* birth defect, *CI* confidence intervals, *OR* odds ratio.

### Prevalence of specific defects by residence

The prevalence of the following specific defects was lower in urban areas than rural areas: cleft lip-palate (3.63 vs. 5.68/10,000, OR = 0.64, 95% CI 0.55–0.74), hydrocephalus (2.79 vs. 4.01/10,000, OR = 0.70, 95% CI 0.59–0.83), cleft lip (2.36 vs. 3.06/10,000, OR = 0.77, 95% CI 0.64–0.94), spina bifida (0.84 vs. 1.39/10,000, OR = 0.60, 95% CI 0.44–0.82), and gastroschisis (0.40 vs. 0.81/10,000, OR = 0.50, 95% CI 0.32–0.77).

The prevalence of the following specific defects was higher in urban than rural areas: congenital heart defect (86.12 vs. 41.07/10,000, OR = 2.11, 95% CI 2.02–2.19), polydactyly (21.99 vs. 17.93/10,000, OR = 1.23, 95% CI 1.14–1.32), other external ear defects (17.47 vs. 12.48/10,000, OR = 1.40, 95% CI 1.29–1.52), syndactyly (7.19 vs. 5.85/10,000, OR = 1.23, 95% CI 1.09–1.39), hypospadias (6.32 vs. 4.61/10,000, OR = 1.37, 95% CI 1.20–1.57), cleft palate (3.24 vs. 2.39/10,000, OR = 1.36, 95% CI 1.13–1.63), anal atresia (3.04 vs. 2.48/10,000, OR = 1.23, 95% CI 1.02–1.48), and Down syndrome (1.74 vs. 1.31, OR = 1.33, 95% CI 1.04–1.71) (Table 8).

**Table 8:** Prevalence of specific defects by residence.

Types	Urban (total = 694,501)		Rural (total = 924,875) (Reference)		OR (95% CI)
	BD (n)	Prevalence (1/10,000, 95% CI)	BD (n)	Prevalence (1/10,000, 95% CI)	
Congenital heart defect	5981	86.12 (83.94–88.30)	3798	41.07 (39.76–42.37)	2.11 (2.02–2.19)
Polydactyly	1527	21.99 (20.88–23.09)	1658	17.93 (17.06–18.79)	1.23 (1.14–1.32)
Other external ear defects	1213	17.47 (16.48–18.45)	1154	12.48 (11.76–13.20)	1.40 (1.29–1.52)
Talipes equinovarus	532	7.66 (7.01–8.31)	694	7.50 (6.95–8.06)	1.02 (0.91–1.14)
Syndactyly	499	7.19 (6.55–7.82)	541	5.85 (5.36–6.34)	1.23 (1.09–1.39)
Hypospadias	439	6.32 (5.73–6.91)	426	4.61 (4.17–5.04)	1.37 (1.20–1.57)
Cleft lip-palate	252	3.63 (3.18–4.08)	525	5.68 (5.19–6.16)	0.64 (0.55–0.74)
Hydrocephalus	194	2.79 (2.40–3.19)	371	4.01 (3.60–4.42)	0.70 (0.59–0.83)
Limb reduction	206	2.97 (2.56–3.37)	302	3.27 (2.90–3.63)	0.91 (0.76–1.08)
Cleft lip	164	2.36 (2.00–2.72)	283	3.06 (2.70–3.42)	0.77 (0.64–0.94)
Cleft palate	225	3.24 (2.82–3.66)	221	2.39 (2.07–2.70)	1.36 (1.13–1.63)
Anal atresia	211	3.04 (2.63–3.45)	229	2.48 (2.16–2.80)	1.23 (1.02–1.48)
Anotia/microtia	169	2.43 (2.07–2.80)	203	2.19 (1.89–2.50)	1.11 (0.90–1.36)
Down syndrome	121	1.74 (1.43–2.05)	121	1.31 (1.08–1.54)	1.33 (1.04–1.71)
Spina bifida	58	0.84 (0.62–1.05)	129	1.39 (1.15–1.64)	0.60 (0.44–0.82)
Diaphragmatic hernia	67	0.96 (0.73–1.20)	95	1.03 (0.82–1.23)	0.94 (0.69–1.28)
Omphalocele	60	0.86 (0.65–1.08)	89	0.96 (0.76–1.16)	0.90 (0.65–1.25)
Esophageal atresia	54	0.78 (0.57–0.98)	54	0.58 (0.43–0.74)	1.33 (0.91–1.94)
Gastroschisis	28	0.40 (0.27–0.58)	75	0.81 (0.63–0.99)	0.50 (0.32–0.77)
Anencephaly	24	0.35 (0.22–0.51)	46	0.50 (0.36–0.66)	0.69 (0.42–1.14)
Encephalocele	16	0.23 (0.14–0.37)	37	0.40 (0.28–0.55)	0.58 (0.32–1.04)
Bladder exstrophy	10	0.14 (0.07–0.26)	14	0.15 (0.08–0.25)	0.95 (0.42–2.14)
Conjoined twins	2	0.03 (0.003–0.10)	3	0.03 (0.006–0.10)	0.89 (0.15–5.31)
Other	5259	75.72 (73.68–77.77)	6051	65.43 (63.78–67.07)	1.16 (1.12–1.20)

*BD* birth defect, *CI* confidence intervals, *OR* odds ratio.

### Prevalence of specific defects by maternal age

The prevalence of congenital heart defects was lower in mothers < 20 than in mothers 25–29 (OR = 0.75, 95% CI 0.62–0.89). In comparison to mothers 25–29, the following specific defects were more frequent in mothers < 20: polydactyly (OR = 1.42, 95% CI 1.13–1.80), talipes equinovarus (OR = 1.60, 95% CI 1.13–2.28), cleft lip-palate (OR = 3.33, 95% CI 2.34–4.74), hydrocephalus (OR = 1.77, 95% CI 1.06–2.93), gastroschisis (OR = 5.80, 95% CI 2.69–12.51) and anencephaly (OR = 6.92, 95% CI 2.81–17.07).

Compared to mothers 25–29, prevalences of the following specific defects were lower in mothers 20–24: congenital heart defect (OR = 0.80, 95% CI 0.75–0.84) and hypospadias (OR = 0.68, 95% CI 0.56–0.83). And the following specific defects were more frequent in mothers 20–24: talipes equinovarus (OR = 1.21, 95% CI 1.05–1.40), cleft lip-palate (OR = 1.78, 95% CI 1.49–2.12), hydrocephalus (OR = 1.41, 95% CI 1.15–1.73), limb reduction (OR = 1.36, 95% CI 1.08–1.70), cleft lip (OR = 1.43, 95% CI 1.12–1.83), gastroschisis (OR = 2.34, 95% CI 1.48–3.68), and encephalocele (OR = 2.53, 95% CI 1.33–4.79).

Compared to mothers 25–29, prevalences of the following specific defects were lower in mothers 30–34: talipes equinovarus (OR = 0.74, 95% CI 0.63–0.87), hydrocephalus (OR = 0.75, 95% CI 0.59–0.95), spina bifida (OR = 0.62, 95% CI 0.40–0.96), and gastroschisis (OR = 0.46, 95% CI 0.22–0.96). And prevalences of the following specific defects were higher in mothers 30–34: congenital heart defect (OR = 1.07, 95% CI 1.02–1.12) and anal atresia (OR = 1.29, 95% CI 1.01–1.63).

Compared to mothers 25–29, mothers ≥ 35 had a higher prevalence of the following specific defects: congenital heart defect (OR = 1.08, 95% CI 1.01–1.15), polydactyly (OR = 1.24, 95% CI 1.11–1.38), cleft lip-palate (OR = 1.47, 95% CI 1.17–1.85), limb reduction (OR = 1.44, 95% CI 1.09–1.90), cleft lip (OR = 1.82, 95% CI 1.38–2.41), anal atresia (OR = 1.87, 95% CI 1.43–2.46), Down syndrome (OR = 3.55, 95% CI 2.55–4.93), diaphragmatic hernia (OR = 2.05, 95% CI 1.32–3.19), omphalocele (OR = 2.16, 95% CI 1.39–3.34), and anencephaly (OR = 2.89, 95% CI 1.52–5.50) (Table 9).

**Table 9:** Prevalence of specific defects by maternal age.

Types	< 20 years old (total = 27,319)			20–24 years old (total = 339,120)			25–29 years old (total = 693,209) (reference)		30–34 years old (total = 385,022)			≥ 35 years old (total = 174,706)		
	BD (n)	Prevalence (1/10,000, 95% CI)	OR (95% CI)	BD (n)	Prevalence (1/10,000, 95% CI)	OR (95% CI)	BD (n)	Prevalence (1/10,000, 95% CI)	BD (n)	Prevalence (1/10,000, 95% CI)	OR (95%CI)	BD (n)	Prevalence (1/10,000, 95% CI)	OR (95% CI)
Congenital heart defect	126	46.12 (38.07–54.18)	0.75 (0.62–0.89)	1669	49.22 (46.85–51.58)	0.80 (0.75–0.84)	4284	61.80 (59.95–63.65)	2537	65.89 (63.33–68.46)	1.07 (1.02–1.12)	1163	66.57 (62.74–70.39)	1.08 (1.01–1.15)
Polydactyly	74	27.09 (20.92–33.26)	1.42 (1.13–1.80)	630	18.58 (17.13–20.03)	0.98 (0.89–1.07)	1320	19.04 (18.01–20.07)	750	19.48 (18.09–20.87)	1.02 (0.94–1.12)	411	23.53 (21.25–25.80)	1.24 (1.11–1.38)
Other external ear defects	43	15.74 (11.38–21.19)	1.06 (0.78–1.44)	492	14.51 (13.23–15.79)	0.98 (0.88–1.09)	1030	14.86 (13.95–15.77)	557	14.47 (13.27–15.67)	0.97 (0.88–1.08)	245	14.02 (12.27–15.78)	0.94 (0.82–1.08)
Talipes equinovarus	33	12.08 (8.31–16.95)	1.60 (1.13–2.28)	310	9.14 (8.12–10.16)	1.21 (1.05–1.40)	522	7.53 (6.88–8.18)	214	5.56 (4.81–6.30)	0.74 (0.63–0.87)	147	8.41 (7.05–9.77)	1.12 (0.93–1.34)
Syndactyly	13	4.76 (2.53–8.16)	0.77 (0.44–1.34)	231	6.81 (5.93–7.69)	1.10 (0.94–1.29)	429	6.19 (5.60–6.77)	246	6.39 (5.59–7.19)	1.03 (0.88–1.21)	121	6.93 (5.69–8.16)	1.12 (0.91–1.37)
Hypospadias	14	5.12 (2.82–8.60)	0.91 (0.54–1.56)	130	3.83 (3.17–4.49)	0.68 (0.56–0.83)	389	5.61 (5.05–6.17)	212	5.51 (4.76–6.25)	0.98 (0.83–1.16)	120	6.87 (5.64–8.10)	1.22 (1.00–1.50)
Cleft lip-palate	35	12.81 (8.89–17.83)	3.33 (2.34–4.74)	232	6.84 (5.96–7.72)	1.78 (1.49–2.12)	267	3.85 (3.39–4.31)	144	3.74 (3.13–4.35)	0.97 (0.79–1.19)	99	5.67 (4.55–6.78)	1.47 (1.17–1.85)
Hydrocephalus	16	5.86 (3.44–9.52)	1.77 (1.06–2.93)	159	4.69 (3.96–5.42)	1.41 (1.15–1.73)	230	3.32 (2.89–3.75)	96	2.49 (1.99–2.99)	0.75 (0.59–0.95)	64	3.66 (2.77–4.56)	1.10 (0.84–1.46)
Limb reduction	5	1.83 (0.59–4.28)	0.67 (0.27–1.62)	126	3.72 (3.07–4.36)	1.36 (1.08–1.70)	190	2.74 (2.35–3.13)	118	3.06 (2.51–3.62)	1.12 (0.89–1.41)	69	3.95 (3.02–4.88)	1.44 (1.09–1.90)
Cleft lip	8	2.93 (1.24–5.78)	1.29 (0.64–2.63)	110	3.24 (2.64–3.85)	1.43 (1.12–1.83)	157	2.26 (1.91–2.62)	100	2.60 (2.09–3.11)	1.15 (0.89–1.47)	72	4.12 (3.17–5.07)	1.82 (1.38–2.41)
Cleft palate	2	0.73 (0.07–2.64)	0.27 (0.07–1.09)	89	2.62 (2.08–3.17)	0.97 (0.75–1.25)	188	2.71 (2.32–3.10)	118	3.06 (2.51–3.62)	1.13 (0.90–1.42)	49	2.80 (2.07–3.71)	1.03 (0.76–1.42)
Anal atresia	9	3.29 (1.46–6.26)	1.42 (0.72–2.78)	79	2.33 (1.82–2.84)	1.00 (0.77–1.31)	161	2.32 (1.96–2.68)	115	2.99 (2.44–3.53)	1.29 (1.01–1.63)	76	4.35 (3.37–5.33)	1.87 (1.43–2.46)
Anotia/microtia	8	2.93 (1.24–5.78)	1.34 (0.66–2.74)	82	2.42 (1.89–2.94)	1.11 (0.85–1.45)	151	2.18 (1.83–2.53)	83	2.16 (1.69–2.62)	0.99 (0.76–1.29)	48	2.75 (2.02–3.64)	1.26 (0.91–1.75)
Down syndrome	4	1.46 (0.37–3.73)	1.35 (0.49–3.70)	42	1.24 (0.89–1.67)	1.14 (0.78–1.67)	75	1.08 (0.84–1.33)	54	1.40 (1.03–1.78)	1.30 (0.91–1.84)	67	3.84 (2.92–4.75)	3.55 (2.55–4.93)
Spina bifida	6	2.20 (0.81–4.80)	1.88 (0.82–4.31)	46	1.36 (0.99–1.81)	1.16 (0.81–1.67)	81	1.17 (0.91–1.42)	28	0.73 (0.48–1.05)	0.62 (0.40–0.96)	26	1.49 (0.97–2.18)	1.27 (0.82–1.98)
Diaphragmatic hernia	2	0.73 (0.07–2.64)	0.87 (0.21–3.58)	29	0.86 (0.57–1.23)	1.02 (0.65–1.60)	58	0.84 (0.62–1.05)	43	1.12 (0.81–1.50)	1.33 (0.90–1.98)	30	1.72 (1.16–2.45)	2.05 (1.32–3.19)
Omphalocele	5	1.83 (0.59–4.28)	2.23 (0.89–5.55)	26	0.77 (0.50–1.12)	0.93 (0.59–1.48)	57	0.82 (0.61–1.04)	30	0.78 (0.52–1.11)	0.95 (0.61–1.47)	31	1.77 (1.20–2.52)	2.16 (1.39–3.34)
Esophageal atresia	2	0.73 (0.07–2.64)	1.30 (0.31–5.39)	23	0.68 (0.43–1.01)	1.21 (0.72–2.02)	39	0.56 (0.40–0.77)	29	0.75 (0.50–1.08)	1.34 (0.83–2.16)	15	0.86 (0.48–1.42)	1.53 (0.84–2.77)
Gastroschisis	8	2.93 (1.24–5.78)	5.80 (2.69–12.51)	40	1.18 (0.84–1.61)	2.34 (1.48–3.68)	35	0.50 (0.35–0.70)	9	0.23 (0.10–0.44)	0.46 (0.22–0.96)	11	0.63 (0.31–1.13)	1.25 (0.63–2.46)
Anencephaly	6	2.20 (0.81–4.80)	6.92 (2.81–17.07)	15	0.44 (0.25–0.73)	1.39 (0.72–2.69)	22	0.32 (0.20–0.48)	11	0.29 (0.14–0.51)	0.90 (0.44–1.86)	16	0.92 (0.54–1.49)	2.89 (1.52–5.50)
Encephalocele	1	0.37 (0.04–2.05)	1.49 (0.20–11.22)	21	0.62 (0.38–0.94)	2.53 (1.33–4.79)	17	0.25 (0.14–0.39)	10	0.26 (0.12–0.48)	1.06 (0.48–2.31)	4	0.23 (0.06–0.58)	0.93 (0.31–2.77)
Bladder exstrophy	1	0.37 (0.04–2.05)	2.54 (0.32–19.82)	4	0.12 (0.03–0.30)	0.82 (0.26–2.61)	10	0.14 (0.07–0.27)	7	0.18 (0.07–0.37)	1.26 (0.48–3.31)	2	0.11 (0.01–0.41)	0.79 (0.17–3.62)
Conjoined twins	0	0.00 (0.00–1.35)	–	2	0.06 (0.006–0.21)	2.04 (0.29–14.51)	2	0.03 (0.003–0.10)	1	0.03 (0.003–0.15)	0.90 (0.08–9.93)	0	0.00 (0.00–0.21)	–
Other	199	72.84 (62.72–82.96)	1.05 (0.91–1.21)	2476	73.01 (70.14–75.89)	1.05 (1.00–1.10)	4832	69.70 (67.74–71.67)	2527	65.63 (63.07–68.19)	0.94 (0.90–0.99)	1276	73.04 (69.03–77.04)	1.05 (0.99–1.12)

*BD* birth defect, *CI* confidence intervals, *OR* odds ratio.

## Discussion

In our study, birth defects were more frequent in males, urban areas, or fetuses of advanced maternal age (≥ 35), and some specific defects were shown to correlate with sex, residence, and maternal age. Based on a representative sample size of the most recent decade of long-term birth defect surveillance data, this study is an important update on the prevalence and epidemiology of birth defects. In this study, we found that the prevalence and epidemiology of some specific defects differed significantly from previous studies, and some other findings rarely mentioned in the previous literature by applying cross-analysis. Therefore, our discovery makes a significant original contribution to the field. In the following discussion, we will examine these differences and discuss the causes in detail.

The overall prevalence of birth defects (188.94/10,000) is consistent with the reported or accepted global prevalence (about 2–3%)^2^. However, there were differences in prevalence between this study and others. e.g. 23.9 per 1000 births in Europe (2003–2007)^22^, 298.6 per 10,000 pregnancies in Japan (2011–2014)^23^, 446.3 per 10,000 births in Korea (2008–2014)^24^, 1.1% of malformed newborns in the Latin American network for congenital malformation surveillance (2017–2019)^25^, 66.2 per 10,000 births in Uganda (2015–2017)^26^, and 184.48 per 10,000 births in India (reported in 2018)^27^. Our prevalence is generally lower than in some high-income countries and higher than in some low- and middle-income countries. There has been no national prevalence of birth defects reported in China recently. Some regions in China reported lower prevalences of birth defects than in this study, such as 13.55 per 1000 births in Guilin, Guangxi Zhuang Autonomous Region (2018–2020)^28^, and 71.51 per 10,000 fetuses in Southern Jiangsu (2014–2018)^29^. We infer that the main reason for these results is differences in diagnosis and reporting rates^30^, as pregnant women had better access to diagnostic and therapeutic services in high-income countries than in low- and middle-income countries. Over the past few decades, there have been significant improvements in diagnostic and therapeutic services for birth defects in China. In addition, birth defects result from hereditary polygenic defects or a gene-environment interaction^13^. Differences in the prevalence of birth defects reported in different studies may also be related to differences in genetic and environmental factors^31–34^. From 2010 to 2020, the prevalence of birth defects decreased. It is inconsistent with previous studies^35, 36^. It may result from many birth defects diagnosed and terminated before 28 weeks of gestation^10^. In our study, these prematurely terminated fetuses are not used to calculate the prevalence of birth defects.

The prevalence of many specific defects appeared to be consistent with the reported or accepted global prevalence, including congenital heart defects, talipes equinovarus, hypospadias, cleft lip-palate, cleft lip, cleft palate, limb reduction, anal atresia, anotia/microtia, omphalocele, and bladder exstrophy^22, 37–47^. However, the prevalences of some specific defects seem to be lower than the reported or accepted global prevalences, such as hydrocephalus (67.5–316.1 per 100,000 births)^48^, Down syndrome (almost 1 in 600 live births)^49^, spina bifida (36.08–243.14 per 100,000 fetuses)^50^, diaphragmatic hernia (1–5 per 10,000 live births)^51^, esophageal atresia (1.9 per 10,000 births in France) ^52^, gastroschisis (2.4/10,000 in Europe, 4.5 per 10,000 live births in the US)^22, 53^, anencephaly (5.5–9.9 per 10,000 births)^54^, encephalocele (global average prevalence is 2.26/10,000)^55^, and conjoined twins (1.32–1.62 per 100,000 births)^56^. Moreover, the prevalences of some specific defects were more than tenfold lower than were generally accepted, such as Down syndrome, encephalocele, and conjoined twins. In contrast, the prevalence of several specific defects was higher than in other studies. E.g., Shin et al. reported lower prevalences of polydactyly and syndactyly in Korea (1.157‰ and 0.309‰)^57^.

We infer that several factors may be related to these differences. First, as discussed, differences in diagnosis and reporting rates may be one of the most critical factors for these results. e.g. with improvements in prenatal screening and diagnosis technologies, most Down syndrome fetuses (which one could argue is not a birth defect but rather a genetic syndrome constituted of numerous co-occurring birth defects) are diagnosed and terminated in the second trimester, resulting in a low prevalence^58^. Second, as is discussed, birth defects may result from hereditary polygenic defects or gene-environment interactions. e.g. polydactyly and syndactyly may be mainly related to chromosomal or genetic abnormalities and are more common in some ethnic groups^59^. Third, the attitudes of pregnant women and their families, affected by the treatment and financial conditions, significantly impact the survival of some fetuses with severe defects, which may also be important factors for these results^60^. Pregnant women in high-income countries are likelier to give birth to babies with defects because there are advanced therapeutic tools and better economic conditions to cure them^61^. Fourth, public health measures were adopted for some specific defects. e.g. the prevalence of neural tube defects (including anencephaly, spina bifida, and encephalocele) has decreased because of the use of folic acid^62^.

Birth defects were more frequent in males, urban areas, or fetuses of advanced maternal age (≥ 35). It is consistent with many previous studies^15, 63, 64^. The following are accepted explanations of this. First, sex differences in the prevalence of birth defects may be related to the male gonad differences during fetal development and the subsequent hormonal and physiologic differences in male and female fetuses^65^. Second, urban–rural differences in the prevalence of birth defects may be related to etiologies, diagnosis, or surveillance, similar to the analysis above^63^. Third, the risk of aneuploidy or non-chromosomal abnormalities increased with maternal age. It is the reason for a higher prevalence of birth defects in fetuses of advanced maternal age^14^. However, several studies have obtained different findings. e.g. very low-quality evidence suggests that advanced maternal age increases the risk of birth defects^19^, low prevalences of major anomalies in advanced maternal age^20^, and a higher prevalence of birth defects in rural areas^21, 66^. It may be partly related to no adjustment of confounders or surveillance methods (such as study populations, surveillance period, and diagnostic methods). In addition, some new features were found in the cross-analysis in this study. First, cross-analysis of sex and residence showed that residence is the main determinant. As discussed, diagnosis and reporting rates may be the main determinant. Second, cross-analysis of sex and maternal age showed that the difference in prevalence between males and females was more significant for maternal age < 20 compared to other age groups. Third, cross-analysis of residence and maternal age showed that the difference in prevalence between urban areas and rural areas is more significant for maternal age 25–34 compared to other age groups. These findings have been rarely covered in previous studies. And the mechanisms are unclear.

Some specific defects were related to maternal age, sex, and residence. Some specific defects were reported in previous studies^63, 67, 68^. However, the epidemiological characteristics of some specific defects differed from other specific defects or previous studies or have rarely been reported. First, cleft palate was the only defect higher in females than males. The possible explanation for this fact may be sex differences in development^69^. Cleft lip-palate and cleft lip were higher in rural than urban areas. There were similar reports^70^. It may be related to material deprivation and parental agricultural work^71, 72^, while the cleft palate is not. Second, some specific defects were more frequent in fetuses of low maternal age, including polydactyly, talipes equinovarus, cleft lip-palate, cleft lip, hydrocephalus, limb reduction, gastroschisis, anencephaly, and encephalocele. There were some similar reports^53, 73–80^, but also there are considerable differences^19^. It may be related to early exposure to risk factors such as tobacco, alcohol, and illicit drugs^19^ and may be easier to influence by confounding factors. Third, some specific defects have rarely been reported. e.g. hydrocephalus was higher in rural than urban areas, which is rarely reported. Jeng et al. found that low socioeconomic status was associated with an increased risk of hydrocephalus, but the reason for this phenomenon is unknown^81^. Fourth, some studies have found different sex characteristics from this study. e.g. Siffel et al. found that bladder exstrophy was almost twice as high in males than in females^47^; Tovar et al. reported a higher prevalence of diaphragmatic hernia in males than females^51^. Fifth, some studies have found different residence characteristics from this study. e.g. several studies found gastroschisis, omphalocele, and anotia/microtia were more frequent in rural areas^45, 82^; Luben et al. found no correlation between residence and spina bifida^83^. Sixth, some studies have found different maternal age characteristics from this study. e.g. several studies reported no correlation between talipes equinovarus, hydrocephalus, and low maternal age^84, 85^; several studies reported higher prevalences of hypospadias and esophageal atresia in fetuses of advanced maternal age^68, 84^. In general, the mechanism for most of the results is unknown. Differences from previous studies and new findings in this study may be related to the location site, sample size, year, or cross-risk factors and bioinformatics testing multi-factors significant level.

The above discussion on the relationship between birth defects and sex, residence, and maternal age suggests that birth defects may result from gene-environment interaction. Although the relationship mechanisms between birth defects and sex, residence, and maternal age were unclear, our study described the phenomenon in detail. It will be beneficial for conducting mechanistic studies in the future.

Some things could be improved. First, data on fetuses under 28 weeks of gestation needed to be included. Second, we did not examine the prevalence of birth defects separately among the live-born or dead infants. Third, some specific defects may be diagnosed after the seventh day, and there is a possibility of underdiagnosing some specific defects, such as congenital heart defects.

## Conclusion

In summary, our data indicate that sex, residence, and maternal age differences in the prevalences of birth defects and most specific defects are common. We have found some new epidemiological characteristics of birth defects using cross-analysis, such as residence is the determining factor for the prevalence of birth defects, the difference in prevalence between males and females was more significant for maternal age < 20 compared to other age groups, the prevalence difference between urban and rural areas is more significant for maternal age 25–34 compared to other age groups. And differences in the epidemiological characteristics of some specific defects from previous studies. Future studies should examine mechanisms. Our findings contributed to clinical counseling and advancing research on the risk factors for birth defects.

## Data Availability

All data generated or analyzed during this study are included in this published article.
